# Supplementary material for: Warming-induced contraction of tropical convection delays and reduces tropical cyclone formation
Source: Nat Commun. 2023 Oct 7;14:6274. doi: 10.1038/s41467-023-41911-5 (PMC10560220; doi:10.1038/s41467-023-41911-5)
Supplement: Supplementary file 1 — Supplementary Information [file 41467_2023_41911_MOESM1_ESM.pdf]

**Supplementary Information for**

# Warming-induced Contraction of Tropical Convection Delays and Reduces Tropical Cyclone Formation


Gan Zhang[1]

[1]Department of Atmospheric Sciences

University of Illinois at Urbana-Champaign

1301 W. Green Street, Urbana, IL 61801

Corresponding Author

Gan Zhang (gzhang13@illinois.edu)

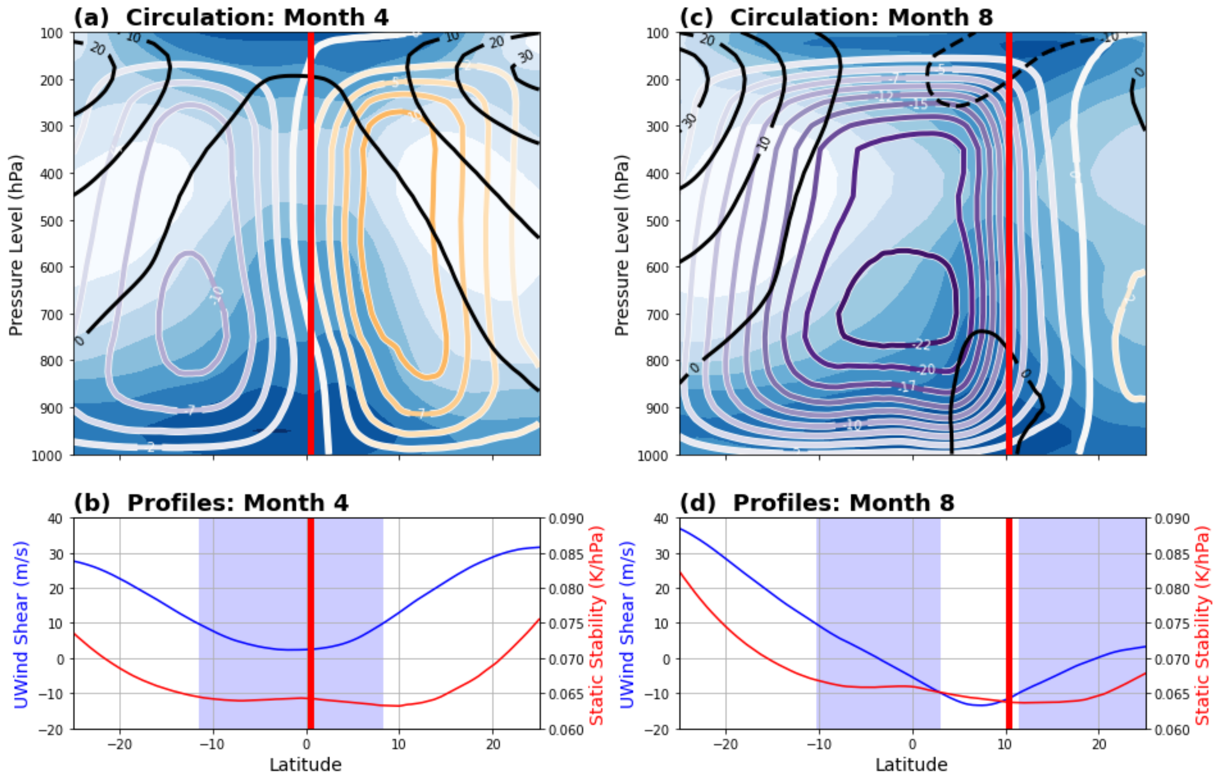


Supplementary Figure 1 Seasonal variations of the Hadley Circulation and the zonally averaged large-scale environment. (a) April climatology (1981-2010) of the meridional mass streamfunction ($10^{10}$ kg $s^{-1}$; orange and purple contours), the zonal wind (m $s^{-1}$), and the relative humidity (%; blue shading). For the streamfunction, the positive values (orange) indicate clockwise motion, and the negative values (purple) indicates counter-clockwise motion. The meridional gradient of the streamfunction is proportional to the strength of vertical motion. The red line marks the latitude of the climatology ITCZ in the corresponding month. (b) April climatology of the vertical shear (blue) of zonal wind (200 hPa – 850 hPa) and the static stability (150 hPa – 850 hPa). The blue shading highlights the latitudes with the magnitude of wind shear smaller than 10 m $s^{-1}$. (c) Same as (a), but for August climatology. (d) Same as (b), but for August climatology.

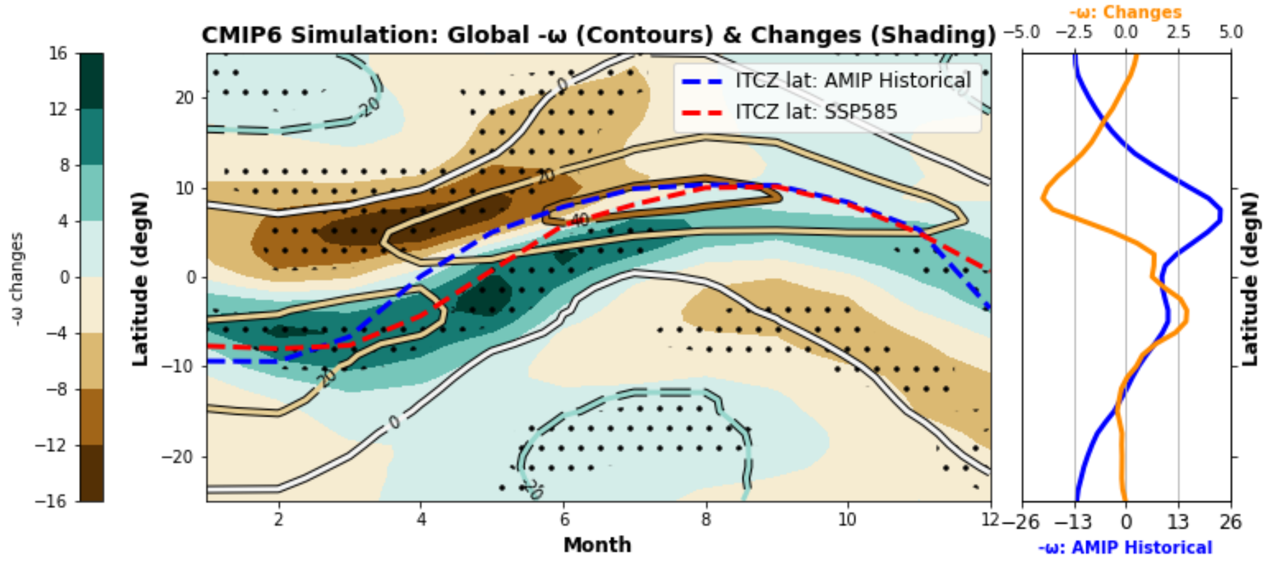


Supplementary Figure 2 The simulated monthly evolution of tropical convection and its changes. Same as Figure 1b, but for the convection changes simulated by twelve selected models of the Coupled Model Intercomparison Project Phase 6 (CMIP6) (see Methods).

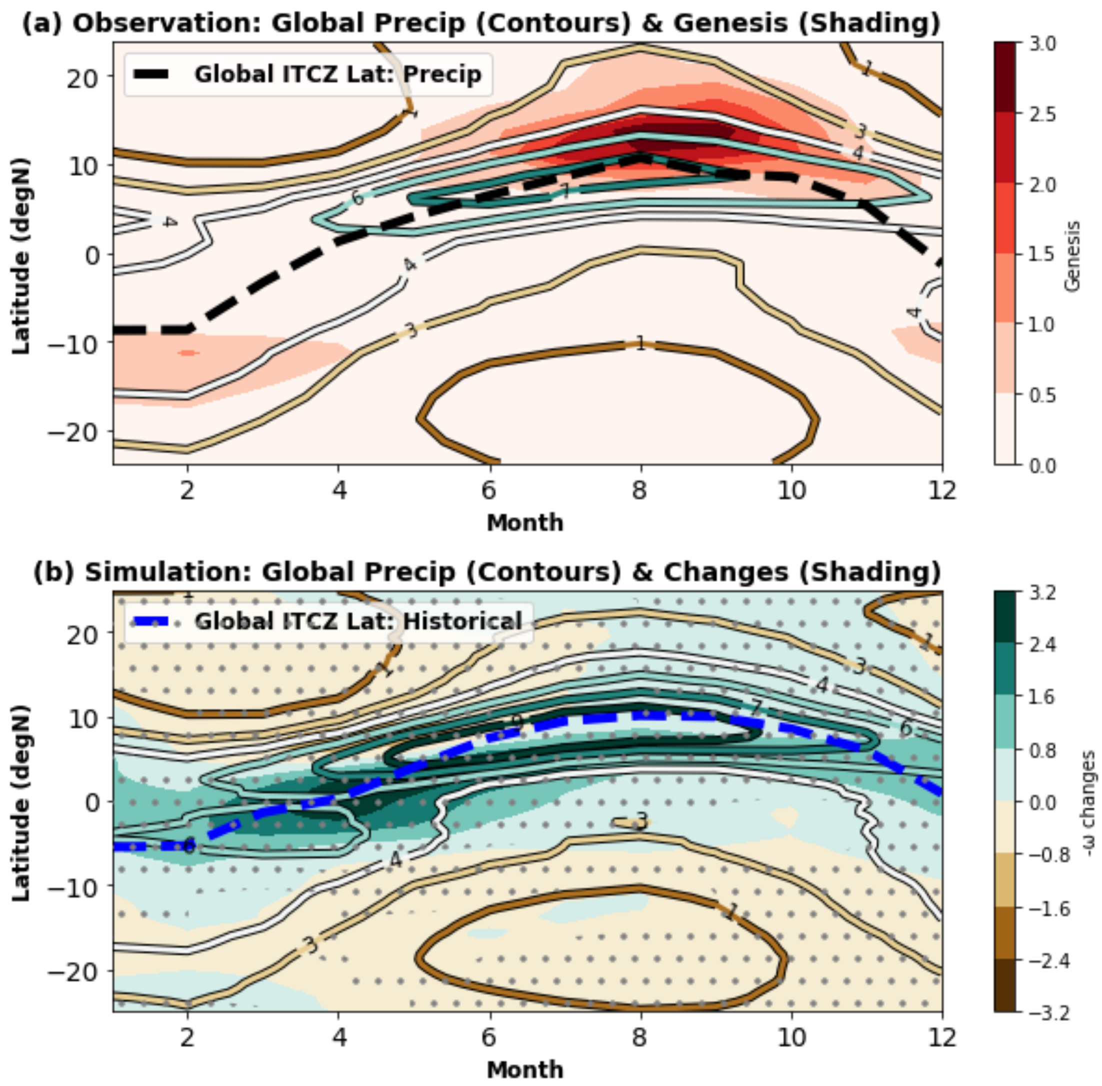


Supplementary Figure 3 The observed and simulated monthly evolution of tropical precipitation and tropical cyclone (TC) genesis frequency. Same as Figure 1a-b, but with the vertical motion replaced with the precipitation (mm day$^{-1}$). The precipitation observation is the 2.5-degree gridded data from the Global Precipitation Climatology Project (GPCP)[1] and available from the National Centers for Environmental Information (NCEI) (https://www.ncei.noaa.gov/data/global-precipitation-climatology-project-gpcp-monthly/).

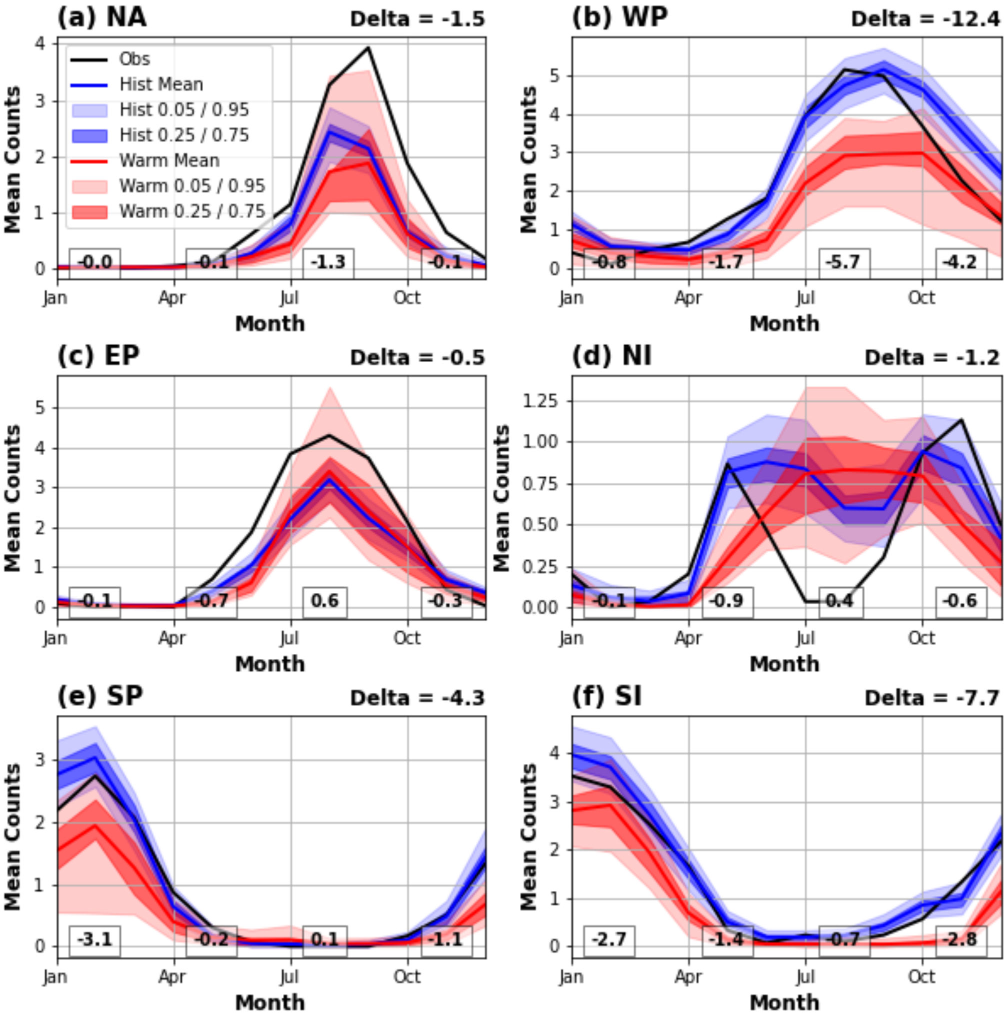


Supplementary Figure 4 Seasonal cycle of tropical cyclone (TC) activity in six basins. The TC frequency ($yr^{-1}$) in (a) North Atlantic, (b) Northwestern Pacific, (c) Northeastern Pacific, (d) North Indian Ocean, (e) South Pacific, and (f) South Indian Ocean is separately evaluated for the historical (blue) and the warming (red) experiments. The light and dark shading indicates the 5$^{th}$-95$^{th}$ percentile range and the 25$^{th}$-75$^{th}$ percentile range. The black solid line shows the observation values during 1981-2010. The changes in the annual TC frequency are denoted in the upper right of subplots. The changes aggregated over three-month periods (Jan-Mar, Apr-Jun, Jul-Sep, and Oct-Dec) are denoted at the bottom of subplots.

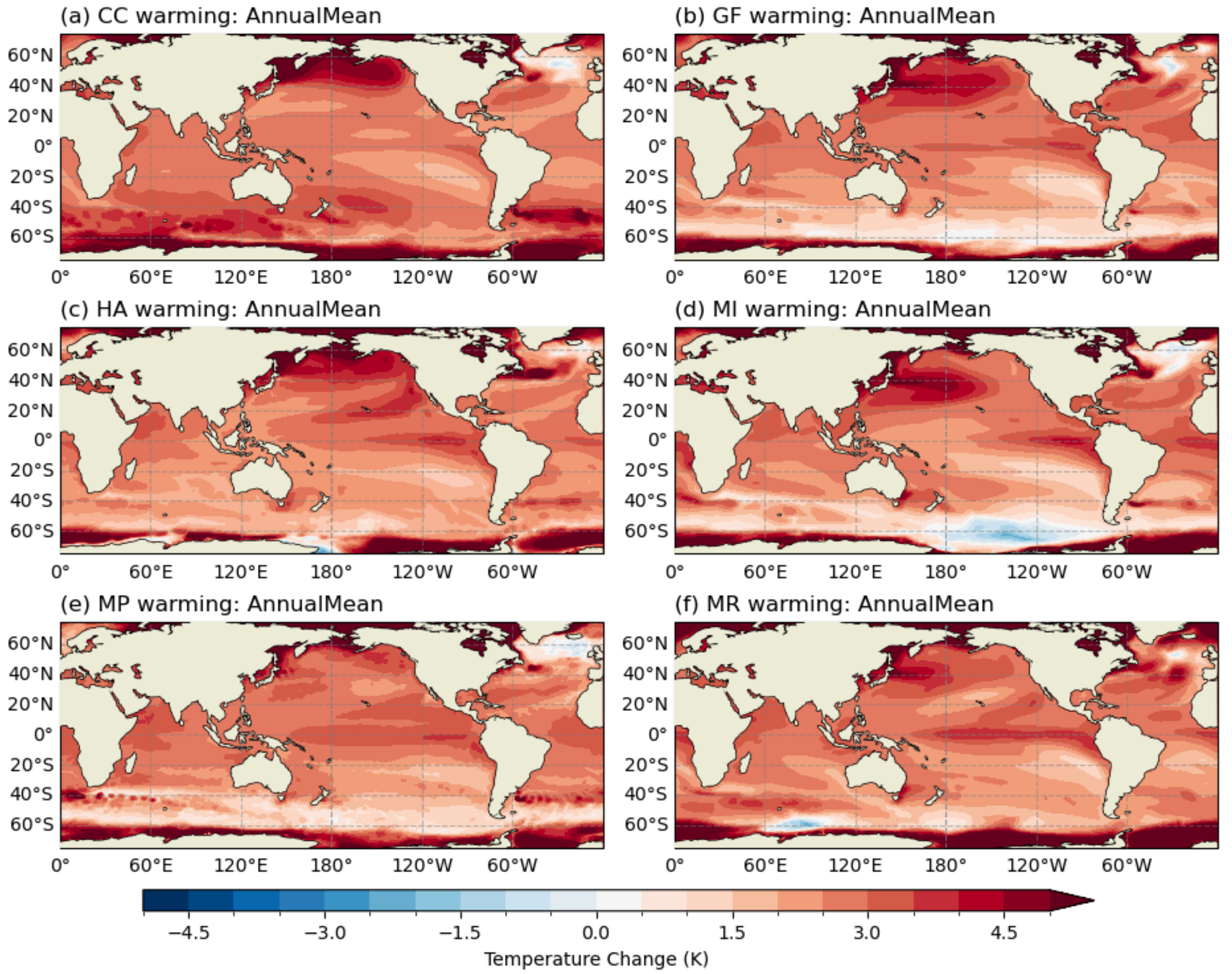


Supplementary Figure 5 Surface temperature differences between the historical (1981-2010) and 4-K warming (2081-2110) simulations. The annual means of seasonally varying patterns that are scaled from the patterns of (a) NCAR-CCSM4 (CC), (b) GFDL-CM3 (GF), (c) HadGEM2-AO (HA), (d) MIROC5 (MI), (e) MPI-ESM-MR (MP), and (f) MRI-CGCM3 (MR). These warming patterns were used to force the large ensemble simulations[2]. The strong warming in polar regions is related to sea ice melting.

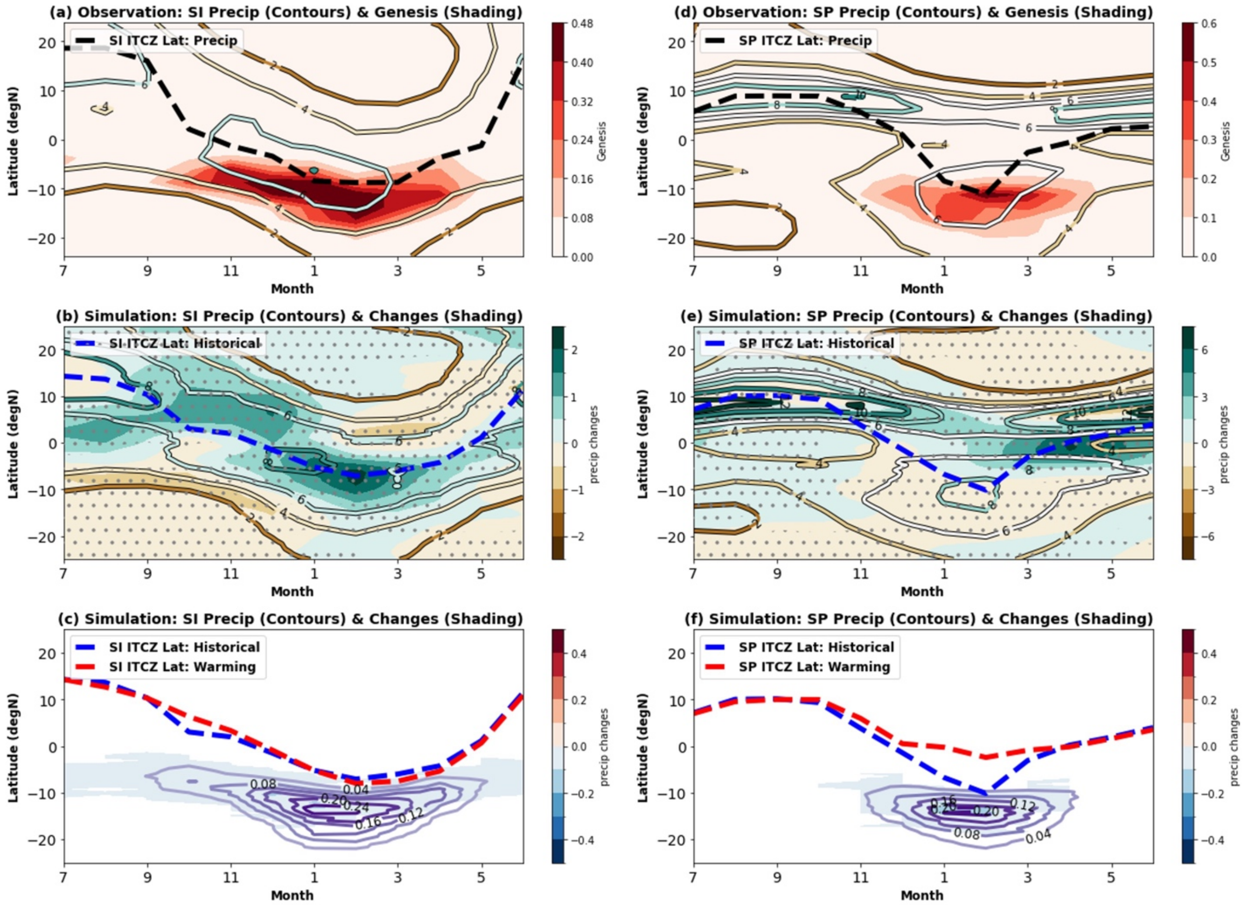


Supplementary Figure 6 The observed and simulated monthly evolution of tropical convection and tropical cyclone (TC) genesis frequency in the Southern Hemisphere basins. Same as Figure 1 but showing the results in (a-b) the South Indian basin and (c-d) the South Pacific basin. The vertical motion is replaced with precipitation (mm day$^{-1}$).

## Supplementary References

1. Adler, R. F. et al. The version 2 Global Precipitation Climatology Project (GPCP) monthly precipitation analysis (1979-present). J. Hydrometeor, **4**, 1147-1167 (2003).

2. Mizuta, R. et al. Over 5,000 Years of Ensemble Future Climate Simulations by 60-km Global and 20-km Regional Atmospheric Models. *Bull. Am. Meteorol. Soc.* **98**, 1383–1398 (2017).
